# Circular RNA circGLIS3 promotes bladder cancer proliferation via the miR-1273f/SKP1/Cyclin D1 axis

**DOI:** 10.1007/s10565-021-09591-3

**Published:** 2021-03-03

**Authors:** Shuilian Wu, Jialei Yang, Haotian Xu, Xin Wang, Ruirui Zhang, Wenmin Lu, Jie Yang, Xiaofei Li, Sixian Chen, Yunfeng Zou, Aruo Nan

**Affiliations:** 1 Department of Toxicology, School of Public Health, Guangxi Medical University, Nanning, 530021 Guangxi China; 2 Guangxi Colleges and Universities Key Laboratory of Prevention and Control of Highly Prevalent Diseases, Guangxi Medical University, Nanning, 530021 Guangxi China; 3 Zhejiang Provincial Key Laboratory of Medical Genetics, Key Laboratory of Laboratory Medicine, Ministry of Education, School of Laboratory Medicine and Life Sciences, Wenzhou Medical University, Wenzhou, 325035 Zhejiang China

**Keywords:** CircGLIS3, MiR-1273f, Bladder cancer, Post-transcriptional regulation

## Abstract

Extensive research confirmed that circRNA can play a regulatory role in various stages of tumors by interacting with various molecules. Identifying the differentially expressed circRNA in bladder cancer and exploring its regulatory mechanism on bladder cancer progression are urgent. In this study, we screened out a circRNA-circGLIS3 with a significant upregulation trend in both bladder cancer tissues and cells. Bioinformatics prediction results showed that circGLIS3 may be involved in multiple tumor-related pathways. Function gain and loss experiments verified circGLIS3 can affect the proliferation, migration, and invasion of bladder cancer cells in vitro. Moreover, silencing circGLIS3 inhibited bladder cancer cell growth in vivo. Subsequent research results indicated circGLIS3 regulated the expression of cyclin D1, a cell cycle–related protein, and cell cycle progression. Mechanically, circGLIS3 upregulates the expression of SKP1 by adsorbing miR-1273f and then promotes cyclin D1 expression, ultimately promoting the proliferation of bladder cancer cells. In summary, our study indicates that circGLIS3 plays an oncogene role in the development of bladder cancer and has potential to be a candidate for bladder cancer.

**Graphical abstract:**

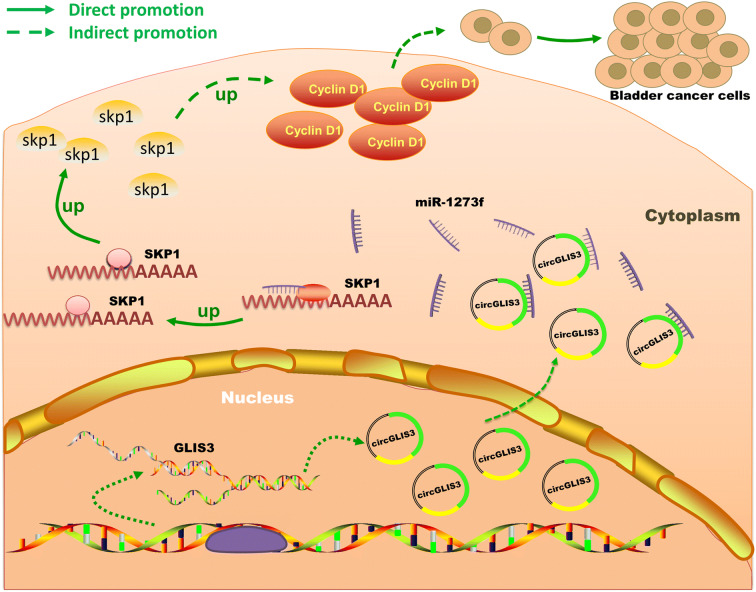

**Supplementary Information:**

The online version contains supplementary material available at 10.1007/s10565-021-09591-3.

## Introduction

Bladder cancer, a malignant tumor, has the highest incidence in the human urinary system. In 2019, there were an estimated 80,470 cases of bladder cancer in the USA, with 17,670 deaths (Siegel et al. 2019). According to the clinicopathologic classification, bladder cancer is primarily divided into two subtypes, including muscular invasive type and non-muscular invasive type. Most patients with non-muscular invasive bladder cancer are treated with surgical resection or combined chemotherapy. However, approximately 50–70% of non-muscular invasive bladder cancer patients will relapse after surgery, and non-muscular invasive bladder cancer in 5–20% of patients will develop into muscular invasive bladder cancer (Knowles and Hurst 2015). At present, although bladder cancer remains difficult to cure (Sun and Trinh 2015; Alfred Witjes et al. 2017), with the rapid development of molecular technology, many diagnostic markers and therapeutic targets have been discovered. Discovery of these molecules will greatly promote the diagnosis and treatment of bladder cancer.

Non-coding RNA has gradually become a popular topic of medical research due to its important biological functions. Several studies have proved that non-coding RNA can participate in the regulation of the pathological process of many malignant cancer (Huang et al. 2016; Liang et al. 2018; Zhang et al. 2019; Yuan et al. 2014). CircRNA has gradually been considered a new type of non-coding RNA, and expression and abundance of circRNA are tissue-specific and cell-specific (Jeck and Sharpless 2014). The closed circular structure of circRNA makes it resistant to exonuclease attenuation and more stable (Li et al. 2015). CircRNAs have been reported to extensively regulate biological functions through post-transcriptional regulation (Hansen et al. 2016). Previously, researches demonstrated circRNA ACVR2A suppresses bladder cancer progression through regulating miR-626/EYA4 axis (Dong et al. 2019); CircRNA INTS4 promotes tumorigenesis in bladder cancer via miR-146b/CARMA3 axis (Zhang et al. 2020). Up to now, it has been widely reported that circRNA adsorbs miRNA to affect the progress of bladder cancer (Cai and Li 2020). In-depth study of circRNA molecules will not only elucidate the complex regulatory networks of non-coding RNA molecules but also provide important guiding significance for the prevention and treatment of malignant tumors.

Excessive proliferation of tumor cells is a major feature of cancer. The proliferation of cancer cells is pathologically caused by the participation of multiple effector genes and many factors (Scatena 2012), but cell cycle is one of the most vital factors regulating proliferation; it can directly promote or inhibit the proliferation process. Cell cycle changes are mainly regulated by two key molecules: cyclin and cyclin-dependent kinases (CDKs) (Nigg 1995). These two regulatory factors can significantly affect the progress of the cell cycle. Cyclin fluctuates regularly during cell cycle turnover, and cyclin D1 is highly expressed in the G1 phase (Men et al. 2018). The length of the cell cycle primarily depends on the G1 phase, and changes in the G1 phase are controlled by cyclin D1. Previous studies showed that circRNA NR3C1 and circRNA 100284 can regulate the cell cycle of bladder cancer cell by inhibiting or promoting cyclin D1 respectively, thereby affecting the progress of bladder cancer (Zheng et al. 2019; Huang et al. 2021). Therefore, elucidating the molecular mechanism that regulates cyclin D1 expression is of considerable significance for understanding the relationship between the cell cycle and the proliferation mechanism of malignant tumors. In addition, S-phase kinase–associated protein 1 (SKP1) is another cell cycle–related protein. Studies have shown that cells with mutated SKP1 expression sequences will stagnate in the G1 or G2 phase, suggesting SKP1 can affect the progress of cell cycle (Bai et al. 1996). SKP1 has been reported to be associated with the progression of bladder cancer (Chen et al. 2016; Wang et al. 2013). Moreover, previous studies demonstrated that SKP1 can regulate the intracellular cyclin-dependent kinase (CDK) expression, especially the cyclin D1 expression (Krajewski et al. 2020; Li et al. 2018a).

CircGLIS3 (called hsa_circ_0002874 in the circBase database) is a 486-bp length circRNA and located at 9p24.2 in the human genome, and its structure forms via circularization of the second exon of the GLIS3 precursor gene. We found for the first time that, compared with normal bladder epithelial cells, circGLIS3 expression was significantly increased in bladder cancer cells through microarray analysis. The expression of circGLIS3 was detected in bladder cancer tissues and adjacent tissues via qPCR (quantitative polymerase chain reaction). And results showed that circGLIS3 expression was also significantly upregulated in bladder cancer tissue, consistent with microarray analysis results. This preliminary result suggests that circGLIS3 may participate in bladder cancer progression. We also used bioinformatics tools to predict the signaling pathways that circGLIS3 might participate in and found that the gene was most abundant in tumor signaling pathways. Then, we confirmed circGLIS3 can promote the progression of bladder cancer through cell function experiments. Further mechanistic experimental studies have indicated that circGLIS3 may competitively bind miR-1273f and promote SKP1 expression, thereby promoting cyclin D1 overexpression, ultimately promoting the proliferation of bladder cancer cells. These above evidences demonstrated that circGLIS3 plays an oncogene role in the development of bladder cancer

## Materials and methods

### Clinical sample collection

The clinical bladder cancer samples used in this study were collected from the Affiliated Hospital of Wen Zhou Medical University. All of the tissues were pathologically diagnosed with urothelial bladder carcinoma. During specimen collection, approximately 3 cm around the bladder cancer tissues was selected as the control of the adjacent tissues. Part of the tissues were fixed with 4% paraformaldehyde (PFA), and the rest was frozen at −80°C for backup. All of the cases included detailed information such as name and pathology. Detail information about clinical bladder cancer samples can be found in supplementary information file 1. The research protocol was approved by the Wenzhou Medical Ethics Committee.

### Laboratory animals

Ten 4-week-old female athymic nude mice were purchased from Shanghai Slack Experimental Animal Center (certificate number SCXK (Su) 201904657). They were raised in the SPF-level experimental area of the Experimental Animal Center of Wenzhou Medical University. Then, they were randomly divided into groups vector and circGLIS3 shRNA. Animals were sacrificed under anesthesia. All of the animal experiments were approved by the Animal Research Committee of Wenzhou Medical University. Animal research was conducted in accordance with international guidelines.

### Nude mouse xenograft model

1 × 10^6^ vector or circGLIS3 shRNA UM-UC-3 stable cell line was inoculated subcutaneously in female athymic nude mice to construct a subcutaneous xenograft tumor model of bladder cancer cells. The weight of the experimental animals was monitored every 3 days. Once the maximum tumor size reached 1000 mm^3^, the animals were sacrificed, and the tumors were dissected for measurement.

### Plasmids and primers

CircGLIS3 overexpressed empty vector (pcircRNA 2.2 hsa) and circGLIS3 stable knockdown empty vector (V40 plvx-shrna2) were purchased from BersinBio Biotechnology Co., Ltd., Guangzhou, China. The overexpressed plasmids and stable knockdown plasmids were homemade. SKP1 overexpressed plasmids and control plasmids were purchased from Hang Seng Technology Co., Ltd., Shanghai, China. CircGLIS3 special plasmids used in the TRAP experiments were purchased from BioChem Technology Co., Ltd., Guangzhou, China. All of the plasmids were prepared using Qiagen plasmid preparation/extraction kits (Valencia, CA, USA). In this study, circGLIS3 siRNA, cyclin D1 siRNA, and all primers were purchased from Gemma Pharmaceutical Technology Co., Ltd., Shanghai, China. Related sequences can be found in Table S1.

### Bladder cancer (BC) cell culture

In this study, normal human urothelial cell lines (SV-HUC-1) and human bladder cancer cell lines (T24, UM-UC-3) were cultured. SV-HUC-1 cells were cultured using F-12k (21127-022, Gibco, USA) containing 10% fetal bovine serum (FBS). T24 cells were cultured in DMEM-F12 (10565-018, Gibco, USA) containing 5% FBS. UM-UC-3 cells were cultured using DMEM medium (11995-065, Gibco, USA) supplemented with 10% FBS. 293 T were cultured in DMEM medium containing 10% FBS.

### RNA isolation, RT-PCR, and qPCR

Total RNA was extracted from BC by using TRIzol reagent, and complementary DNA (cDNA) was synthesized from the total RNA according to the reverse transcription kit instructions. A GoTaq qPCR kit was used for quantitative PCR (qPCR).

### Cell transfection

Plasmids were transfected in vitro with PolyJet transfection reagent (SignaGen Laboratories, Gaithersburg, MD, USA) and siRNA was transfected with RiboFect CP (RiboBio, Guangzhou, China). All transfection experiments were conducted following the manufacturer’s instructions. Transfection efficiency was determined via qPCR after 48 h.

### Stably transfected cell lines were screened

Using the 239 T plasmid packing lentivirus tool, the virus was infected after suspension. Fresh medium was replaced after 12 h. After 24 h, the status and density of the cells were assessed. If the cell density was appropriate for digestion, the cells were diluted into single cell suspensions. The single cell suspension was seeded into 96-well plates. Fluorescence microscopy was used to observe the cell extraction monoclonal batches.

### Soft agar

This experiment evaluated the ability of tumor cells to anchor independent growth. Medium and agar were added to a 50-mL centrifuge tube at a ratio of 3:2 and mixed thoroughly, and then, 1.2 mL agar and 1.8 mL medium were added to each hole in the tube. The solution could set for 4 h at room temperature. A cell suspension was prepared, diluted 2–5 times according to the cell volume, the number of cells was counted, and the cell suspension liquid volume was calculated. First, 736 mL of medium was added to an EP tube, and then, 264 mL of agar was added with a sample gun to mix it. Finally, a 10,000-cell suspension was added. After the top glue solidified at room temperature, a sealing strip was affixed and cultured in a CO_2_ incubator at 37°C for 7–15 days for observation and photography.

### EdU assay

An EdU detection kit was used to detect the cell proliferation ability. After seeding the cells into a 96-well plate, they were cultured in an incubator. The cells were treated separately according to the grouping described in the text. The EdU solution was diluted with the complete cell culture medium at a ratio of 1000:1 to prepare an appropriate amount of 50 μM EdU medium. Subsequent steps were strictly in accordance with the instructions of Cell-Light EdU Apollo567 In Vitro Kit (RiboBio, Guangzhou, China).

### Western blot protein analysis

Cell lysis buffer (10 mM Tris-HCl, pH 7.4, 1% SDS, and 1 mM Na_3_VO_4_) was used to prepare whole cell extracts, and the proteins were extracted from the cultured cells in each group following the manufacturer’s instructions. The protein concentration was detected by a BioDrop microanalyzer. The same amount of protein was separated by SDS-PAGE electrophoresis, then transferred to a membrane, and blocked at room temperature for 1 h with 5% skim milk powder. Cyclin D1 (1:1000), Cyclin E (1:1000), Cyclin A (1:1000), Cyclin B (1:1000), SKP1 (1:1000), α-Tubulin (1:4000), and GAPDH (1:4000) antibodies were added and incubated at 4°C overnight. Goat anti-rabbit and mouse IgG secondary antibody was added and incubated at 4°C for 3 h. Images were obtained.

### CCK-8 (Cell Counting Kit-8) assay

Cell vitality was detected via a CCK-8 detection kit. After seeding the cells in a 96-well plate, they were cultured in an incubator. The cells were treated separately as described in the text, and 100 μL of CCK-8 solution was added to each well and incubated for 2 h, and the optical density (OD) at a wavelength of 450 nm was measured.

### Flow cytometry to detect the cycle of BC cells

The cell cycle was detected on a flow cytometer using an FITC cycle detection kit (KGI). After processing according to the instructions, the cells were collected, centrifuged, and incubated at a density of 1 × 10^6^/mL. Then, 100 μL of cell suspension was incubated with 10 μL annexin V FITC for 15 min. After counter-staining with PI in the dark for 30 min, the cells were analyzed by flow cytometry.

### Cell apoptosis

An annexin V FITC/PI apoptosis assay kit KGA106 (KeyGen, Nanjing, China) was used to stain the cells and CytoFlex flow cytometry was used to detect apoptosis. The ratio of early apoptotic cells to late apoptotic cells was calculated.

### Transwell assay

Approximately 3 × 10^4^ cells were inoculated in 0.1% FBS medium in a transwell chamber (353097, Corning, NY, USA) for 24 h. Invasion measurements were made according to the manufacturer’s instructions. Cells were inoculated in 400 mL of 0.1% culture medium in the upper compartment, and 700 mL of complete culture medium was added to the lower compartment. The cell invasion time was 24 h. The cells were fixed with 3.7% formalin for 5 min at room temperature, washed with PBS, and let stand in 100% methanol for 20 min. The cells were washed with PBS and stained at room temperature with Giemsa (diluted 1:1 in PBS) for 15 min in the dark. The cells on the upper surface of the membrane were washed with PBS, and the cells on the lower surface of the membrane were erased. Five locations were randomly selected using an optical microscope (DMi1) to count the cells stained on the submembrane.

### Tagged RNA affinity purification (TRAP)

According to TRAP experimental instructions (BersinBio Biotechnology, Guangzhou, China), plasmids and control plasmids connected with circGLIS3 sequences were transfected into target cells. After the cells were overgrown, they were collected and lysed, and circGLIS3 was captured by magnetic beads coated with probes. MicroRNA bound to circGLIS3 was also precipitated. The microRNA was collected and purified for reverse transcription.

### Statistical analysis

All of the data are from at least three independent experiments and presented as mean ± standard deviation (SD). The difference significance between different groups was analyzed by the 2-tailed *t* test. *P* < 0.05 was considered statistically significant.

## Results

### CircGLIS3 expression is upregulated in bladder cancer

**Fig. 1:**
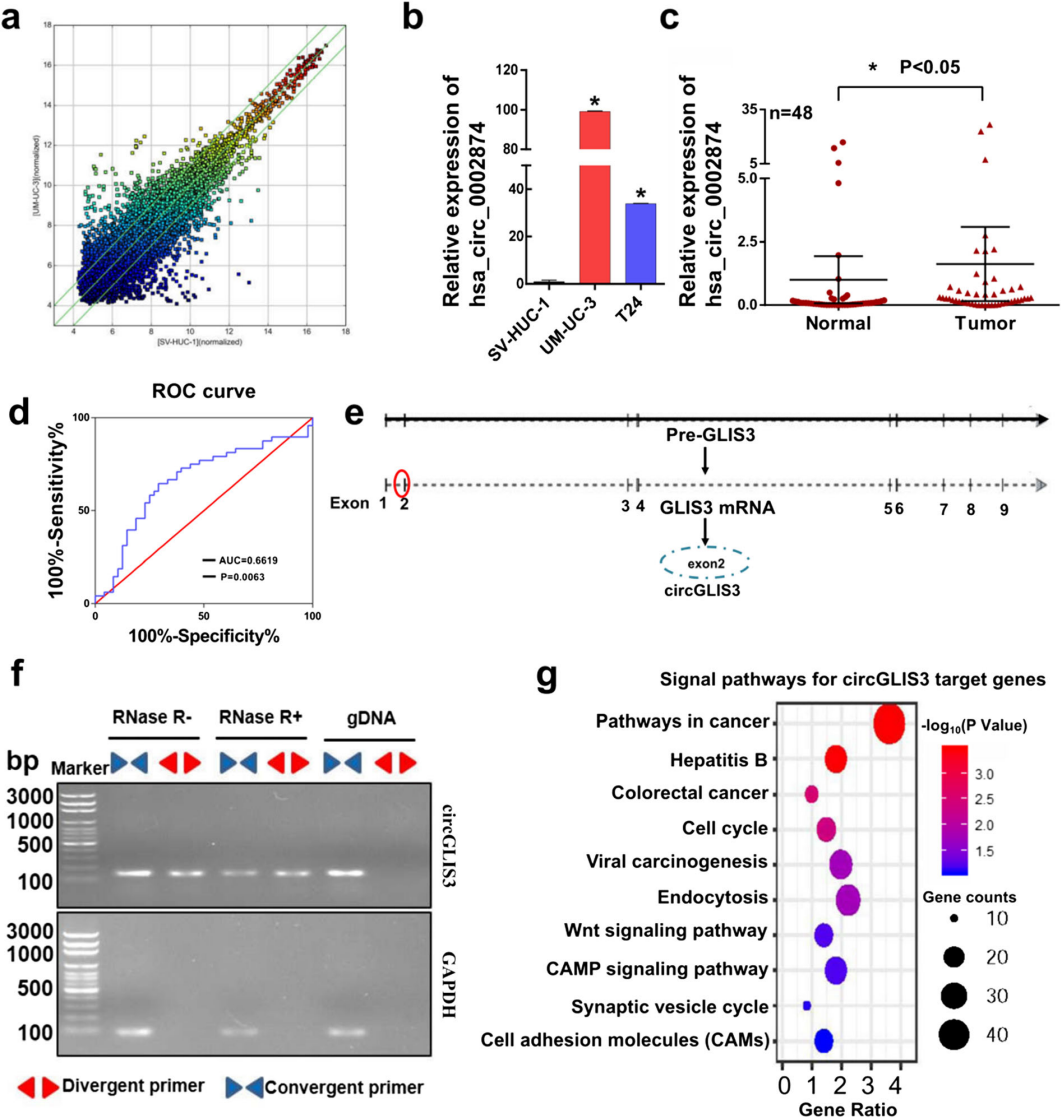
CircGLIS3 is upregulated in bladder cancer cells and bladder cancer tissues. **a** Using microarray gene chip analysis, circRNAs with the most significant differences were screened from normal bladder epithelial cells (SV-HUC-1) and bladder cancer cells (UM-UC-3). **b** The relative expression of has_circ_0002874 in bladder cancer cell lines UM-UC-3, T24, and normal bladder epithelial cells was detected using qPCR technology. **c** The expression level of hsa_circ_0002874 in bladder cancer tissues and adjacent tissues was detected by qPCR, and the symbol (*) indicates statistical significance (*P* < 0.05). **d** ROC curve analysis was used to evaluate the diagnostic value of hsa_circ_0002874. The area under the curve was 0.6619 (*P* < 0.05). **e** The genomic structure of circGLIS3. **f** The circular structure of circGLIS3 was verified by agarose gel electrophoresis. **g** KEGG analysis of aggregation in various signal pathways of downstream target genes of circGLIS3. The symbol (*) indicates statistical significance (*P* < 0.05)

With the rapid development of high-throughput technology and research into non-coding RNA, many non-coding RNAs have been explored for their biological functions in different cancers. Among them, circRNA, as a newly emerging non-coding RNA, is gradually becoming well known. We used circRNA microarray technology to screen a pair sample of normal bladder epithelial cells (SV-HUC-1) and bladder cancer cells (UM-UC-3). As shown in Fig. 1a and Supplementary Fig. S1a, there were a total of 60 upregulated genes and 77 downregulated genes. CircRNA microarray dataset in this study has been deposited to GEO DataSets (https://www.ncbi.nlm.nih.gov/gds); accession number is GSE159239. All detailed raw data can be found in this circRNA microarray dataset. The most significant difference was hsa_circ_0002874 (69-fold upregulation). We verified the eight circRNAs that were most significantly upregulated in the circRNA differential expression profile using qPCR (Table S1; Fig. S1b). We found hsa_circ_0002874 was most significantly increased in the bladder cancer cells. We used qPCR to detect the relative hsa_circ_0002874 expression in SV-HUC-1, UM-UC-3, and T24 (bladder cancer cells). Result showed that hsa_circ_0002874 expression was increased 99.2 and 33.8 times in UM-UC-3 cells and T24 cells, respectively (Fig. 1b). To verify whether this differential expression is also present in bladder cancer patients, we collected bladder cancer tissues and adjacent tissues from 48 patients and detected the relative hsa_circ_0002874 expression using qPCR (Fig. 1c). Compared with the adjacent tissues, hsa_circ_0002874 was upregulated in the bladder cancer tissues (*P* < 0.05). Combined with the ROC curve analysis results, the area under the curve is 0.662, suggesting that hsa_circ_0002874 may have some diagnostic value in bladder cancer (Fig. 1d). Therefore, we speculate that the upregulation of hsa_circ_0002874 likely promotes the development of bladder cancer.

To verify the circular structure of hsa_circ_0002874, we analyzed the sequence of hsa_circ_0002874 and found that hsa_circ_0002874 is located on chromosome 9 with the gene symbol GLIS3. According to its gene symbol, it was called circGLIS3. CircGLIS3 is 486 bp in length and is formed by circularizing exon 2 (Fig. 1e). Based on the sequence and structural characteristics of circGLIS3, we designed three pairs of primers to verify the structure of circGLIS3. The first pair was designed at the circular first junction of circGLIS3 (divergent primer), which can specifically amplify circular RNA; the second pair was designed at the junction of the second and third exons (convergent primer). This primer can specifically amplify GLIS3 linear transcripts. The third pair was designed at the junction of the second exon and the intron. The extracted RNA was divided into an RNase R-treated group and an untreated group for reverse transcription, and genomic DNA of UM-UC-3 cells was extracted. The two sets of cDNA and genomic DNA were used as PCR templates for amplification. After the RNase R treatment, the amplified product of the convergent primer was significantly weakened, while the amplified product of the divergent primer was basically unchanged. The product of genomic DNA as a template had only linear bands and no circular bands, demonstrating that circGLIS3 has a circle structure (Fig. 1f). To study how circGLIS3 functions in the progression of bladder cancer, we used regRNA (http://regrna2.mbc.nctu.edu.tw/) and TargetScan (http://www.targetscan.org/) website respectively to predict the miRNAs that circRNA may bind to and the downstream target genes of these miRNAs. Then, KEGG pathway analysis was conducted by DAVID (https://david.ncifcrf.gov/) for classifying and aggregating the downstream target genes of circGLIS3. The highest number of genes aggregated on the pathway in cancer, which indicated that circGLIS3 has considerable potential to play a role in the cancer pathway (Fig. 1g).

### CircGLIS3 significantly promotes the proliferation, invasion, and migration of bladder cancer cells

**Fig. 2:**
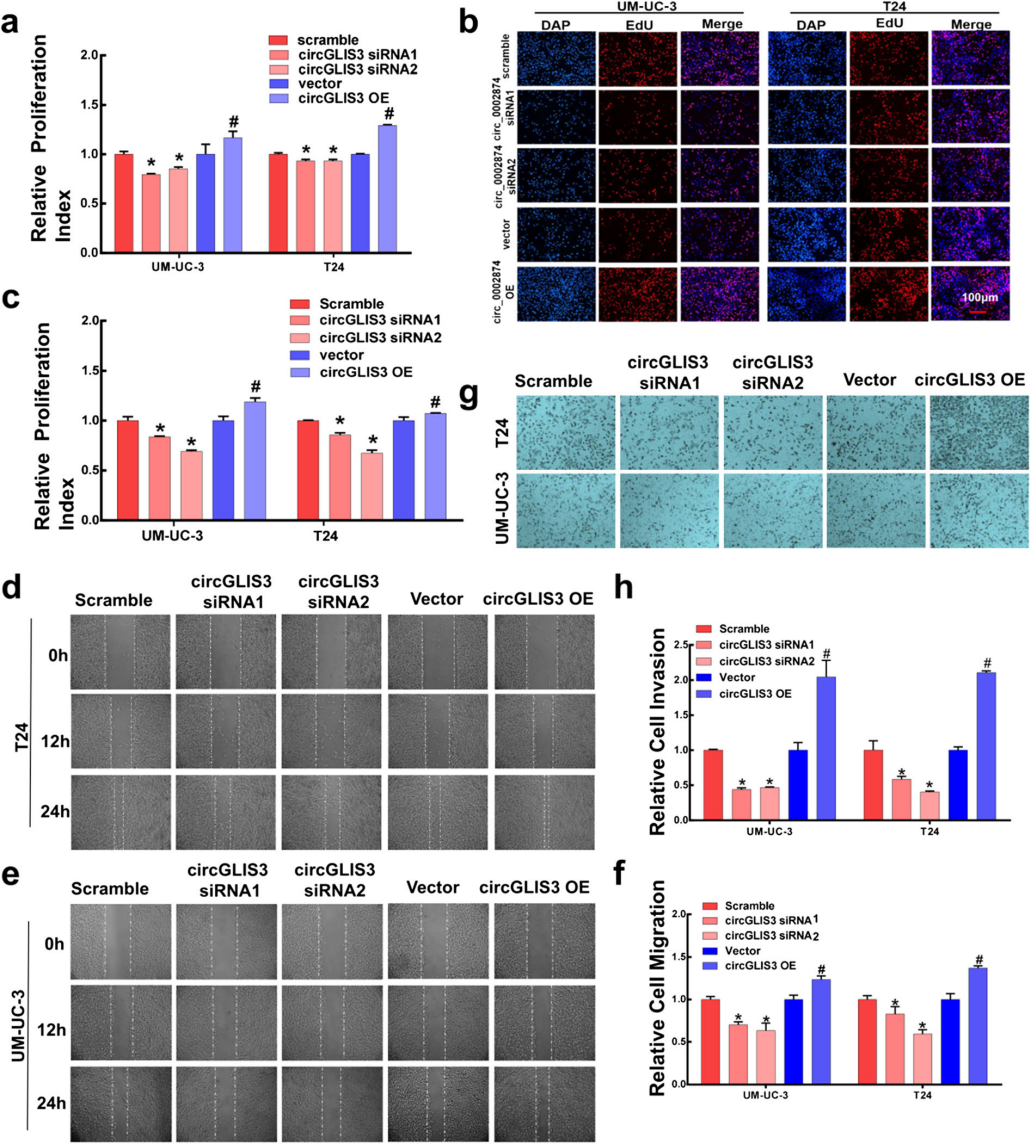
CircGLIS3 can promote the proliferation and invasion of bladder cancer cells. **a** CCK-8 assay measured the effects of circGLIS3 transient silence and overexpression on cell viability. **b**, **c** The effects of circGLIS3 silencing and overexpression on cell proliferation were detected by EdU experiments. Each bar indicates the mean ± SD of three independent experiments. OE stands for overexpression, which means that a gene is overexpressed. **d**, **e** UM-UC-3 and T24 cells were transiently silenced and overexpressed by circGLIS3. **f** The migration of UM-UC-3 cells was photographed at 0 h, 12 h, and 24 h. T24 cells were observed at 0 h, 12 h, and 24 h. Images of the migration. **g**, **h** UM-UC-3 and T24 cells were transiently silenced and overexpressed by circGLIS3. After 48 h, the cells were digested, resuspended, seeded into an invasion chamber, stained, and photographed after 24 h. The symbol (*) indicates statistical significance compared to scramble group (*P* < 0.05), The symbol (#) indicates statistical significance compared to vector group (*P* < 0.05)

Based on the sequence of circGLIS3, we designed two circGLIS3 siRNAs and constructed an overexpressed plasmid of circGLIS3 (Fig. S1c). The knockdown or overexpression efficiency was verified by transient transfection in two bladder cancer cells (UM-UC-3 and T24) (Fig. S1d). Second, CCK-8 assay, EdU test, scratch test, and transwell were used to detect the proliferation, invasion, and migration of the two bladder cancer cells after transient transfection (Fig. 2a–h). Knocking down the expression of circGLIS3 significantly inhibited the proliferation, invasion, and migration of bladder cancer. Increasing the expression level of circGLIS3 can significantly promote the proliferation, invasion, and migration ability of bladder cancer cells. These results showed that circGLIS3 can significantly promote the proliferation, invasion, and migration of bladder cancer cells.

### CircGLIS3 promotes bladder cancer cell growth in vivo

**Fig. 3:**
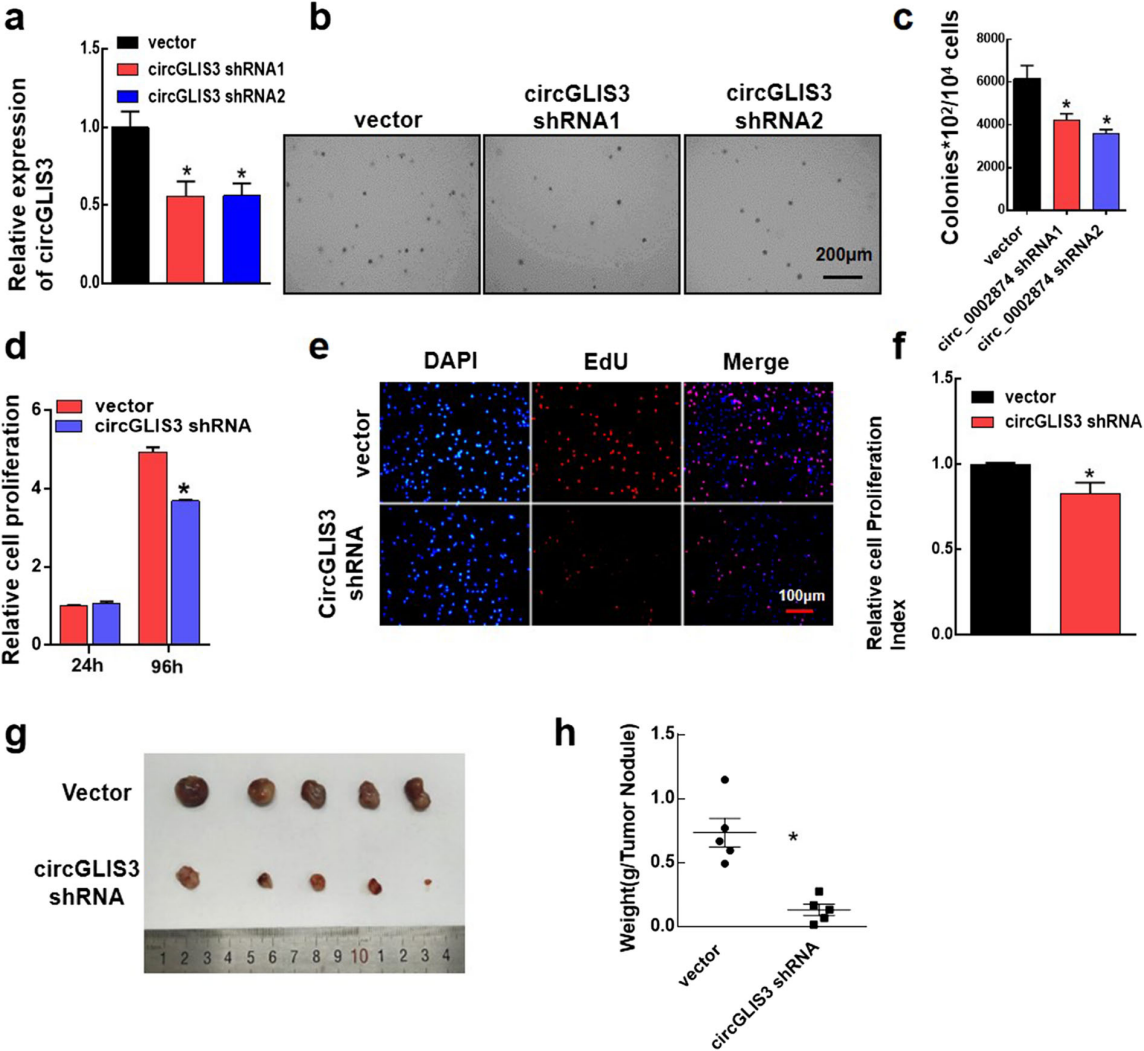
CircGLIS3 significantly promotes the proliferation of bladder cancer cells in vivo. **a** The relative expression levels of UM-UC-3 stably transfected cell lines (vector, circGLIS3 shRNA1, and circGLIS3 shRNA2) were detected via qPCR. **b**, **c** Approximately 10,000 cells of three stably transfected cell lines of vector, circGLIS3 shRNA1, and circGLIS3 shRNA2 were seeded in soft agar in a six-well plate and photographed after 15 days of culture. **d** The cell viability of stably transfected cell lines (vector and circGLIS3 shRNA) at 24 h and 96 h was detected using the CCK-8 assay. **e**, **f** An EdU test was used to detect the proliferation of stably transfected cell lines (vector and circGLIS3 shRNA) after 48 h. **g**, **h** Subcutaneous tumor tissue was removed from nude mice on day 28 of subcutaneous injection of cells. The subcutaneous tumor tissues of the two groups were photographed and weighed. The symbol (*) indicates statistical significance compared to scramble group (*P* < 0.05)

We conducted nude mouse experiments to explore whether circGLIS3 plays the same biological role in the in vivo environment. We used UM-UC-3 cells to construct a stable knockdown of circGLIS3 cell lines (circGLIS3 vector, circGLIS3 shRNA1, circGLIS3 shRNA2) and verified their efficiency (Fig. 3a). Soft agar colony formation is a good indicator for monitoring cell proliferation. We inoculated three stable cell lines into soft agar, respectively, and photographed the lines after 15 days of growth. After knocking down the expression level of circGLIS3, the cell colony forming ability was significantly reduced (Fig. 3b, c). We selected a cell line that stably knocked down circGLIS3 to verify the transient results (CCK-8 and EdU). Knocking down the expression level of circGLIS3 can inhibit bladder cancer cell proliferation, which was consistent with the transient results (Fig. 3d–f).

The stable transformed cell lines were used as the research subject. Four-week-old BALB/c nude mice were randomly divided into two groups, and two groups of stable transformed cell lines were injected subcutaneously on the right back, each mice with 2.5 × 10^6^ cells. The status of the mice was assessed and recorded every week. The mice were sacrificed 4 weeks after the injection, and the tumor tissues under their skin were removed and recorded (Fig. 3g, h). After knockdown circGLIS3 expression, the subcutaneous tumor tissue appeared later, and the tumor tissue was smaller and lighter. These results showed that circGLIS3 can significantly promote bladder cancer cell line growth in vivo.

### CircGLIS3 promotes cell G0/G1 phase progression via cyclin D1

**Fig. 4:**
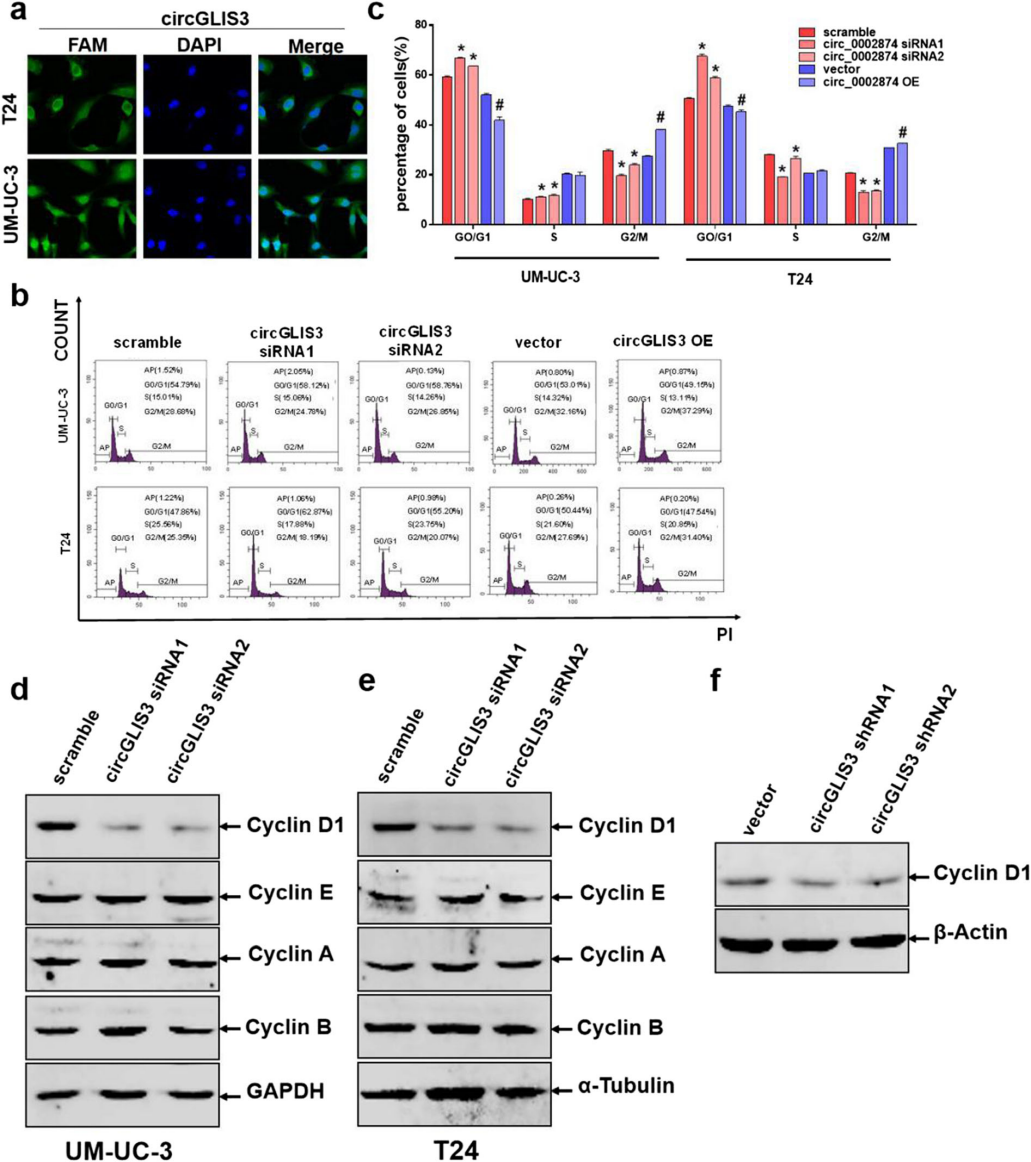
CircGLIS3 promotes cell G0/G1 phase progression via cyclin D1. **a** The distribution of circGLIS3 in T24 and UM-UC-3 cells was determined by FISH. **b**, **c** The effects of circGLIS3 transient silence and overexpression on the cell cycle were detected using flow cytometry. **d**, **e** Western blot was used to detect the expression of Cyclin D1, Cyclin E, Cyclin A, and Cyclin B in transiently transfected bladder cancer cells. **f** The expression of cyclin D1 in stably transfected cell lines (vector, circGLIS3 shRNA1, and circGLIS3 shRNA2) was detected via Western blot. The symbol (*) indicates statistical significance compared to scramble group (*P* < 0.05), The symbol (#) indicates statistical significance compared to vector group (*P* < 0.05)

To explore the relevant mechanism that circGLIS3 plays in promoting the proliferation of bladder cancer cells, we determined its localization in UM-UC-3 cells and T24 cells via fluorescence in situ hybridization (Fig. 4a). Results showed that circGLIS3 was mainly distributed in the cytoplasm. We then explored the mechanism by which circGLIS3 promotes the proliferation of bladder cancer cells. First, we transiently silenced and overexpressed circGLIS3 in bladder cancer cell lines (UM-UC-3 and T24) and harvested the cells 48 h after transfection. Second, after staining the cells with PI, flow cytometry was used to detect the cell cycle. Knocking down the expression level of circGLIS3 led to G0/G1 phase arrest, thereby inhibiting the proliferation of bladder cancer cells, while upregulating the expression level of circGLIS3 significantly inhibited G0/G1 phase arrest, thus promoting bladder cancer cell proliferation. This result revealed that circGLIS3 may regulate the proliferation level of bladder cancer by affecting the cell cycle (Fig. 4b, c).

At present, the cell cycle is generally divided into four phases: pre-DNA synthesis (G1), DNA synthesis (S), late DNA synthesis (G2), and division (M). Corresponding regulatory proteins, such as Cyclin C, D, and E, are the major regulatory proteins in the G1 phase, Cyclin A is the major regulator in the S phase, and Cyclin B is the major regulatory protein in the G2M phase. We used previously mentioned experiments to determine whether circGLIS3 can affect the cycle change of bladder cancer cells. To study the molecular mechanism of the G0/1 blockade of the cell cycle caused by knocking down circGLIS3, we tested the relevant regulatory molecule Cyclin D1 that affects the cell cycle G0/G1 transition. First, we transiently transfected circGLIS3 siRNA (transient silencing) on bladder cancer cells (UM-UC-3 and T24). Second, we used a stable circGLIS3 knockdown cell line for verification. After knocking down the expression level of circGLIS3, the expression of Cyclin D1 considerably decreased, while the expression of Cyclin E, Cyclin A, and Cyclin B did not change significantly, suggesting that circGLIS3 may affect cell cycle changes by regulating the expression of Cyclin D1, thereby promoting bladder cancer cell proliferation (Fig. 4d–f).

### SKP1 mediates circGLIS3 regulation on cyclin D1 expression

**Fig. 5:**
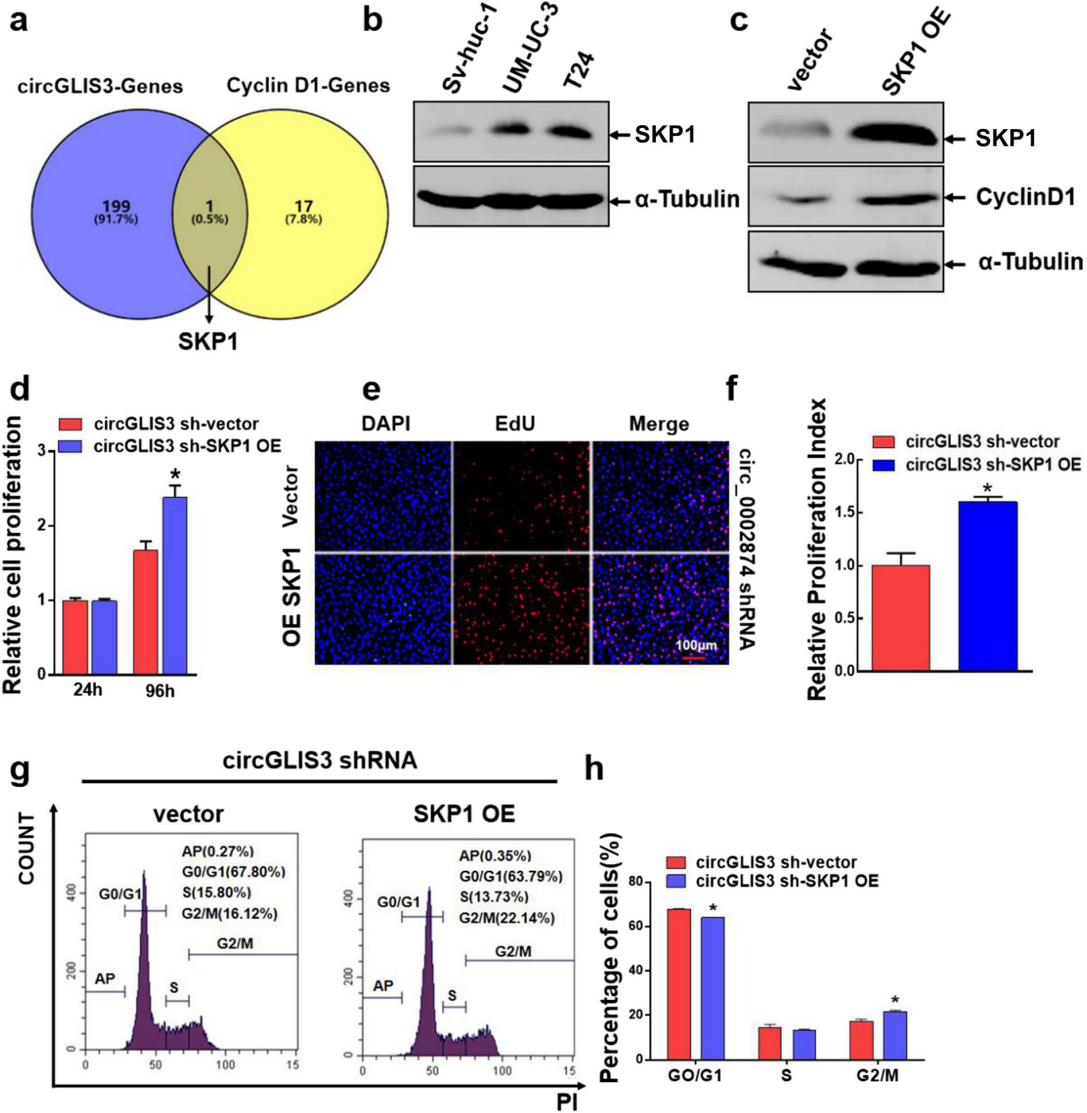
SKP1 mediates circGLIS3 regulation of Cyclin D1 expression. **a** Prediction of downstream target genes (a total of 199) of circGLIS3 using bioinformatics methods, and prediction of upstream target genes of Cyclin D1 (a total of 17) via KEGG analysis. **b** The protein level of SKP1 in SV-HUC-1, UM-UC-3, and T24 cells was detected using Western blot. **c** SKP1 was overexpressed in circGLIS3 shRNA stably transfected cell lines (UM-UC-3), and protein expression of SPK1 and Cyclin D1 was detected via Western blot. **d** CCK-8 assays were conducted to detect the cell viability of circGLIS3 shRNA stably transfected cell lines transfected with vector and SKP1 overexpressed plasmids for 24 h and 96 h. **e**, **f** An EdU test was conducted to detect the cell proliferation of circGLIS3 shRNA stably transfected cell lines 48 h after transfection of vector and SKP1 overexpressed plasmids. **g**, **h** SKP1 was transiently overexpressed in circGLIS3 shRNA stably transfected cell lines, and the Cycle status of the vector and SKP1 OE groups was detected via flow cytometry. The symbol (*) indicates statistical significance compared to vector group (*P* < 0.05)

To explore how circGLIS3 regulates Cyclin D1, we used bioinformatics to predict 199 downstream genes of circGLIS3 related to proliferation. We used KEGG analysis to screen 17 upstream genes of Cyclin D1 and intersect them. As shown in Fig. 5a, only SKP1 was both the downstream gene of circGLIS3 and the upstream gene of Cyclin D1. To verify this, we first used Western blot to detect SKP1 expression in normal bladder epithelial cells (SV-HUC-1) and bladder cancer cells (UM-UC-3 and T24) (Fig. 5b). Compared with SV-HUC-1, SKP1 expression was increased in UM-UC-3 and T24, suggesting that SKP1 may participate in the development of bladder cancer. Second, we transiently overexpressed SKP1 in the circGLIS3 shRNA stable cell line. The expression levels of both SKP1 and Cyclin D1 increased (Fig. 5c). This result confirms that, during the bladder cancer process, SKP1 can regulate the expression of Cyclin D1.

SKP1 has been predicted to be a downstream gene of circGLIS3 and an upstream gene of Cyclin D1. To determine whether SKP1 participates in the function of circGLIS3, we transiently overexpressed SKP1 in circGLIS3 shRNA stable cell lines and performed CCK-8 assay. The cell activity was detected at 24 h and 96 h (Fig. 5d). And the proliferative activity of bladder cancer cells was detected via an EdU test for 48 h (Fig. 5e, f). After the expression level of SKP1 increased, the proliferative activity was significantly promoted, indicating that SKP1 can enhance the proliferative activity of bladder cancer and reverse cell proliferation inhibition caused by circGLIS3 shRNA.

To further determine whether the SKP1 pathway promotes the proliferation of bladder cancer cells consistent with circGLIS3-Cyclin D1, we transiently overexpressed SKP1 in circGLIS3 shRNA stable cell lines and used flow cytometry to detect the cycle change of bladder cancer cells (Fig. 5g, h). Compared with the vector group, the G0/G1 phase arrest of the SKP1 OE group cells was significantly inhibited. This result suggests that the overexpressed SKP1 relieves G0/G1 phase block caused by circGLIS3 shRNA, thereby enhancing the proliferative activity of bladder cancer cells.

### CircGLIS3 may regulate SKP1 expression via miR-1273f

**Fig. 6:**
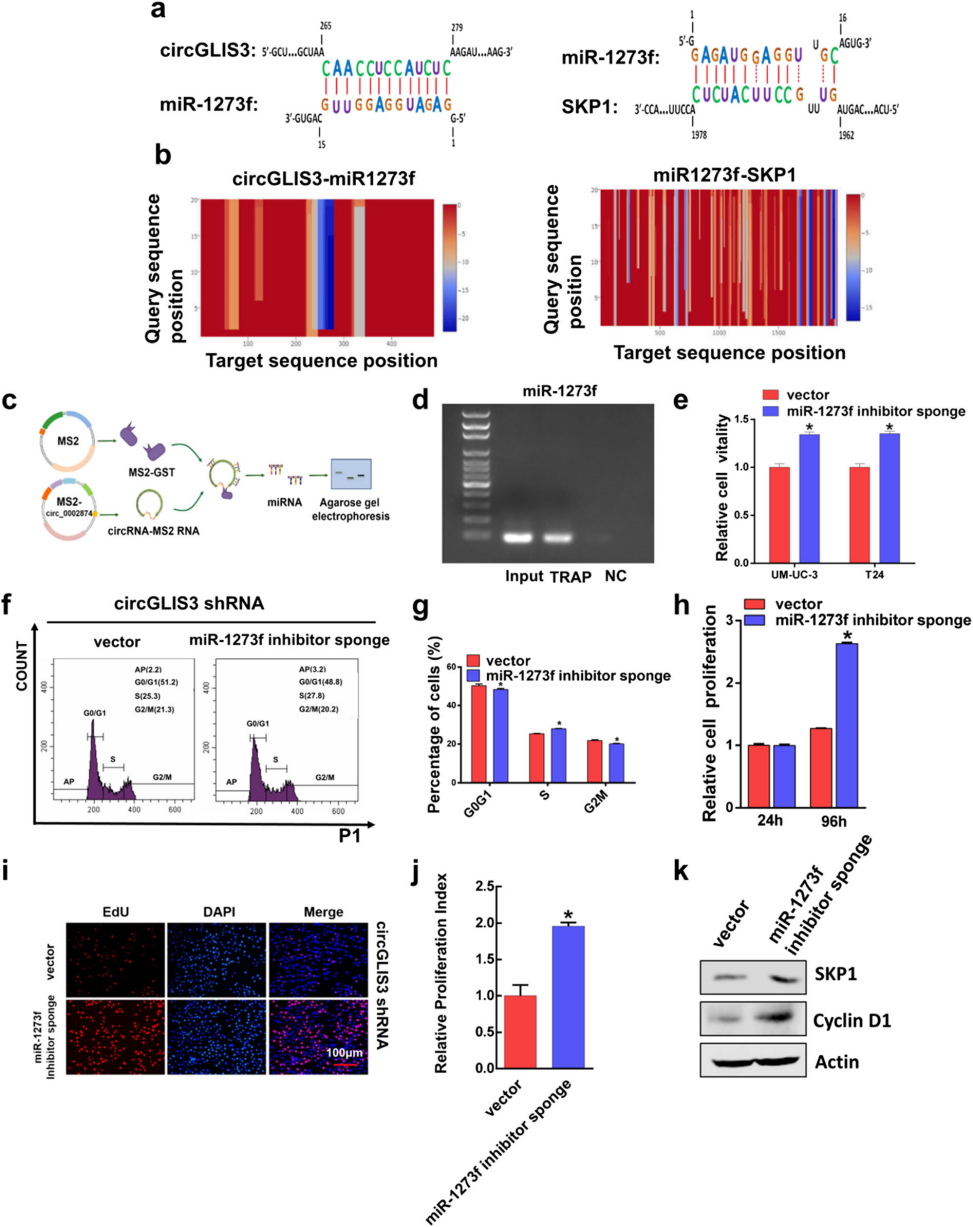
CircGLIS3 may regulate SKP1 expression via miR-1273f. **a** Prediction of potential target genes of circGLIS3 through the bioinformatics website. MiR-1273f had the highest matching sequence with circGLIS3, and the miR-1273f seed sequence matched SKP1. **b** Heat map analysis of miR-1273f binding to circGLIS3 and SKP1 sequences. **c** Schematic of the TRAP experiment. **d** Specific microRNA directly binding to circGLIS3 was pulled down, and the expression of miR-1273f in the input, TRAP, and NC groups was detected using agarose gel electrophoresis. **e** The analysis of single knockdown of miR-1273f on proliferation of bladder cancer cell. **f**, **g** In the UM-UC-3 cell line that stably knocked down the expression of circGLIS3, knocking down miR-1273f expression can inhibit the arrest of bladder cancer cell G0/G1 phase. **h** Stably knock down the expression of circGLIS3 and miR-1273f in the UM-UC-3 cell line, and detect the cell proliferation changes at 24 h and 96 h by ATP experiment. **i**, **j** The EdU experiment was used to detect changes in cell proliferation after simultaneously knocking down the expression of miR-1273f in cell lines that stably knocked down circGLIS3. **k** The changes of SKP1 and cyclin D1 protein in the cell lines stably knocking down the expression of circGLIS3 and miR-1273f were detected by WB experiment. The symbol (*) indicates statistical significance compared to vector group (*P* < 0.05)

To explore the underlying mechanism of circGLIS3 regulating SKP1, we screened a batch of microRNAs with a high matching degree to circGLIS3 by the regRNA website (http://regrna2.mbc.nctu.edu.tw/). Among them, miR-1273f had the highest matching degree with circGLIS3, and the seed sequence of miR-1273f matched SKP1 was also the highest matching degree. A schematic of the base matching is shown in Fig. 6a, and the combined heat map is shown in Fig. 6b. The predicted results suggested that circGLIS3 may adsorb miR-1273f like a sponge, so that the binding of miR-1273f to SKP1 decreases, thereby promoting the expression of SKP1 and increasing the expression of Cyclin D1, and ultimately promoting the proliferation of bladder cancer cells. To determine whether miR-1273f is a direct downstream target gene of circGLIS3, we designed a specific circGLIS3 TRAP experimental probe. Via a TRAP experiment, we used the circGLIS3 specific probe to pull down circGLIS3 and microRNA (Fig. 6c). Primers for miR-1273f were designed, and the TRAP experiment product was used as a template for PCR amplification. The amplified product was used for agarose gel electrophoresis (Fig. 6d). Significantly amplified bands and almost no product amplification in the NC group suggested that miR-1273f can directly bind to circGLIS3.

To further explore the role of miR-1273f in the regulation of bladder cancer proliferation by circGLIS3, firstly, we knocked down miR-1273f expression by miR-1273f inhibitor and found that decreased miR-1273f promoted the proliferation of bladder cancer cells (Fig. 6e). Then, we stably knocked down miR-1273f expression in cell lines that have stably knocked down circGLIS3 and constructed a knockdown of circGLIS3 and miR-1273f stable cell line. Exploring changes in the proliferation of bladder cancer cells on this cell line, it was found that knocking down miR-1273f could counteract the effect of simply knocking down circGLIS3. Stable knockdown of miR-1273f expression can effectively inhibit G0/G1 phase arrest caused by knocking down circGLIS3 (Fig. 6f, g) and promote the proliferation of bladder cancer cells (Fig. 6h–j) and promote the expression of SKP1 and cyclin D1 (Fig. 6k). This result suggests that miR-1273f not only participates in circGLIS3 to regulate bladder cancer cell proliferation but also regulates SKP1 and cyclin D1 expression.

## Discussion

Bladder cancer is one of the most common malignant tumors of the urinary system. According to statistics from 2018, the global incidence of bladder cancer ranks twelfth among all malignant tumors, while the latest Chinese research shows that the incidence of bladder cancer in China ranks seventh (Bray et al. 2018). The most common pathological type of bladder cancer is urothelial carcinoma (Chen et al. 2019). Most patients’ first diagnosis is superficial bladder cancer, while a few are diagnosed with muscle-infiltrating bladder cancer (Alfred Witjes et al. 2017). Among them, the treatment of non-muscular infiltrating bladder cancer is mainly local resection assisted by intravesical BCG or chemotherapy as the first choice, but patients undergoing this treatment have a high risk of recurrence. Muscle-infiltrated bladder cancer is mainly treated with a combination of surgery, chemotherapy, and radiotherapy, and approximately half of patients will recur within 2 years, and some will have distant metastasis when they recur. Although researchers have made some progress in the study of bladder cancer, such as identifying a wide variety of tumor markers and therapeutic targets (Bazrafshani et al. 2016; Noel et al. 2015; Puntoni et al. 2016; Onal et al. 2015), the treatment of bladder cancer is still not optimistic. Therefore, new and effective tumor markers and drug targets are urgently needed to support clinical diagnosis and treatment, and research into novel target molecules is extremely important.

CircGLIS3 is a type of endogenous circular non-coding RNA molecule. Compared with traditional linear RNA molecules, circRNA is not only more stable in structure but also may be more advantageous in terms of widespread distribution and sequence conservation (Jeck et al. 2013; Salzman et al. 2012; Chao et al. 1998; Zhang et al. 2014; Zhang et al. 2013). Recent studies demonstrated that circRNA is closely related to the pathological processes of various malignant tumors such as lung cancer, gastric cancer, liver cancer, pancreatic cancer, and bladder cancer (Bachmayr-Heyda et al. 2015; Liu et al. 2018; Tang et al. 2018; Song et al. 2019; Cai et al. 2018; Li et al. 2018b). Through microarray analysis and subsequent qPCR detection, we found for the first time that circGLIS3 expression was significantly upregulated in bladder cancer tissues and bladder cancer cells. But ROC curve analysis showed AUC of circGLIS3 was 0.66, which indeed indicated that it might have some diagnostic value but not an excellent biomarker.

So far, the most widely studied function of circRNA is to regulate downstream target gene expression by adsorbing microRNA. MicroRNA is a class of endogenous non-coding small RNA approximately 22nt in length, which can bind to the 3′UTR of the target mRNA molecule and degrade target mRNA, thereby regulating target mRNA expression (Bartel 2004; Alvarez-Garcia and Miska 2005). Previously, circular RNA circ-ZKSCAN1 was reported to inhibit bladder cancer progression by adsorbing miRNA (Bi et al. 2019). And Liu et al. (2020) found that hsa_circ_0001361 promotes bladder cancer progress by the miR-491-5p/MMP9 axis. Su et al. (2020) reported that circRIP2 promotes bladder cancer progression by the miR-1305/Tgf-β2/smad3 axis. These evidences indicated that circRNAs acting as an adsorption sponge to adsorb miRNAs to regulate downstream target gene expression and pathways play a pivotal role in bladder cancer progression. In this study, we firstly revealed the interaction between circGLIS3 and miR-1273f in bladder cancer. Sequence comparisons showed that the seed sequence of miR-1273f matched the 3′UTR region of SKP1, so SKP1 was probably the downstream target gene of miR-1273f. Subsequent experiments in this study confirmed miR-1273f can bind to the 3′UTR region of SKP1. As an important skeletal protein, the complex formed by SKP1 plays an important role in a variety of malignant tumors. However, there have been few reports to date on SKP1’s effect on bladder cancer. In this study, we ascertained that the SKP1 level of protein expression in bladder cancer cells increased significantly. We also found that increases in the expression of SKP1 can reverse cell proliferation inhibition caused by knockdown circGLIS3. These results suggest that SKP1 is a downstream target molecule of circGLIS3 and participates in proliferation regulation of bladder cancer cells.

Cyclin D1 is a cell cyclin encoded by the CCND1 gene, with a length of 12 kb and located at 11q13. Its content in cells changes periodically, which regulate the transformation of cells from the G1 to S phase (Qie and Diehl 2016). Previously, cyclin D1 was reported to be abnormal in a variety of cancers and is closely related to the abnormal proliferation of tumor cells (Wang et al. 2014). And cyclin D1 overexpression will lead to abnormal cell proliferation and even malignant transformation. Here, we reported a new mechanism for the regulation of cyclin D1. CircGLIS3 acts as a sponge to absorb miR-1273f, thereby reducing the degradation of SKP1 by miR-1273f, relatively upregulating SKP1 expression. Increased SKP1 expression would further promote the expression of cyclin D1, ultimately leading to an abnormally excessive proliferation of bladder cancer cells. However, the molecular mechanism that SKP1 promotes the upregulation of cyclin D1 expression remains unclear, and further investigation is needed. We also found through preliminary exploration that circGLIS3 can promote the migration of bladder cancer, but the specific mechanism must be further studied. Nevertheless, these findings increase the understanding of the molecular mechanism of bladder cancer cell proliferation and preliminarily elucidate the significance of circGLIS3 in the development of bladder cancer.

**Fig. 7:**
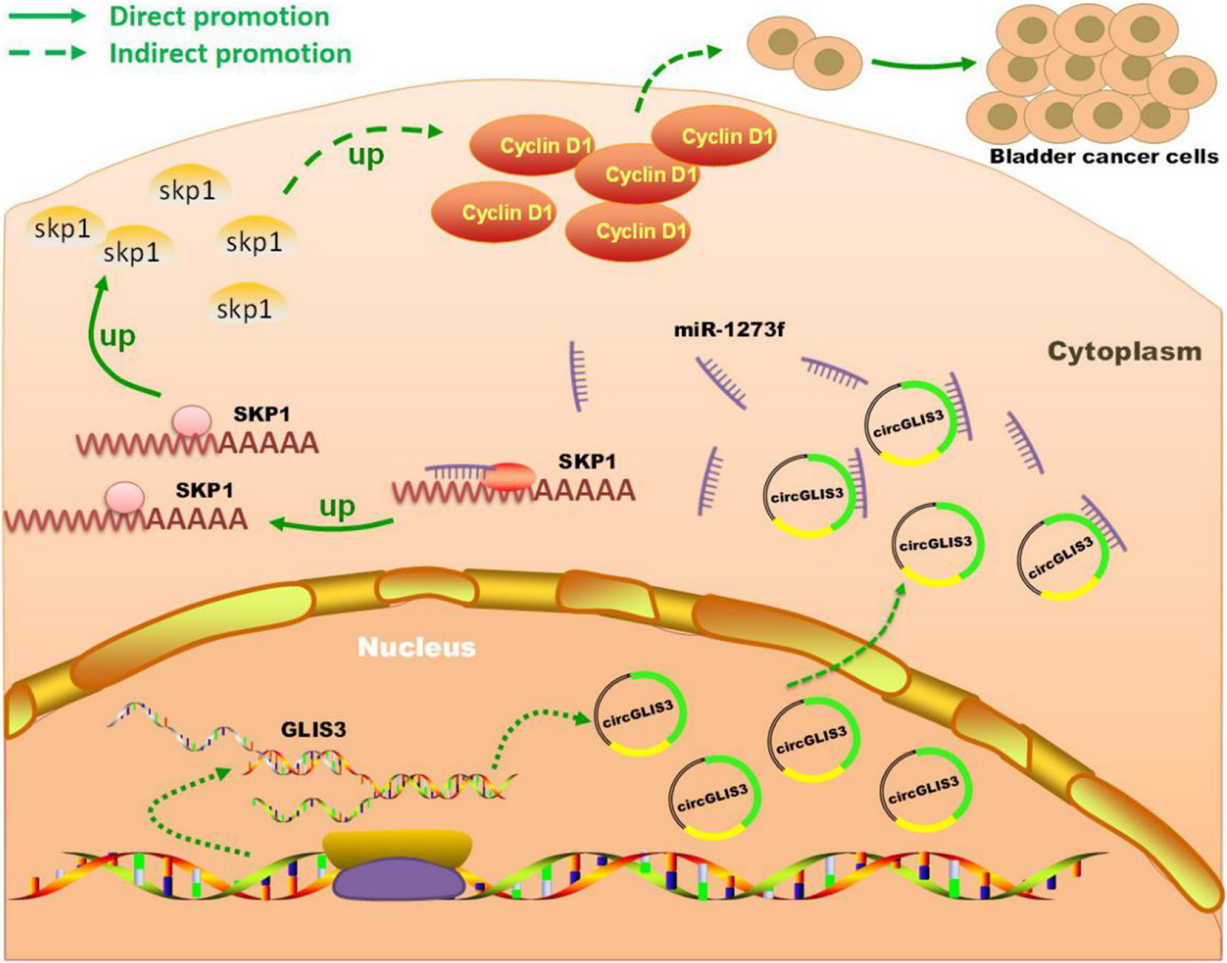
Schematic summary of the mechanism by which circGLIS3 promotes the proliferation of bladder cancer cells

In conclusion, this study found that circGLIS3 affect the progress of bladder cancer cells in vitro, and silencing circGLIS3 inhibited bladder cancer growth in vivo. Mechanically, circGLIS3 upregulates the expression of SKP1 by adsorbing miR-1273f and then promotes the expression of cyclin D1, ultimately promoting bladder cancer cell proliferation (Fig. 7). These results will be further validated through in vivo experiments. This study not only improves the understanding of circRNA molecules but also demonstrates a potential molecular target for bladder cancer, providing a new candidate for bladder cancer treatment and the development of targeted drugs.

## Supplementary information

Electronic supplementary material can be found in supplementary information file.

ESM 1 (XLS 44 kb)

ESM 2 (DOCX 125 kb)

## Data Availability

CircRNA microarray dataset in this study has been deposited in the Gene Expression Omnibus (GEO) DataSets (https://www.ncbi.nlm.nih.gov/gds) under the following accession numbers: GSE159239. Other data and materials used and/or analyzed during the current study are available from the corresponding authors on reasonable request.
