# Supplementary material for: Circular RNA circGLIS3 promotes bladder cancer proliferation via the miR-1273f/SKP1/Cyclin D1 axis
Source: Cell Biol Toxicol. 2021 Mar 3;38(1):129–46. doi: 10.1007/s10565-021-09591-3 (PMC8789643; doi:10.1007/s10565-021-09591-3)
Supplement: Supplementary file 2 — (DOCX 125 kb) [file 10565_2021_9591_MOESM2_ESM.docx]

**Supplementary Table and Figure**

**Table S1 Information of the qPCR primer** **sequences and silencing RNA sequences**

| **qPCR primer name** | **Sequence (5’-3’)** |
| --- | --- |
| circGLIS3 (Forward) | AGCAGCAGGAGTTTGGAAGC |
| circGLIS3 (Reverse) | AAAACCTGTGGCCAAGACGG |
| SKP1 (Forward) | ATGCCTTCAATTAAGTTGCAG |
| SKP1 (Reverse) | CCCAGTTCCTCTACCAAATG |
| CyclinD1 (Forward) | CTGTCCTACTACCGCCTCAC |
| CyclinD1 (Reverse) | ACCTCCTCCTCCTCCTCTTC |
| GAPDH (Forward) | ATCAATGGAAATCCCATCACCA |
| GAPDH (Reverse) | GACTCCACGACGTACTCAGCG |
| **siRNA name** | **Sequence (5’-3’)** |
| circGLIS3 siRNA1 (sense) | CCUGGGAAAGGCUUAUAACTT |
| circGLIS3 siRNA1 (antisense) | GUUAUAAGCCUUUCCCAGGTT |
| circGLIS3 siRNA2 (sense)  circGLIS3 siRNA2 (antisense)  scramble (sense)  scramble (antisense)  **shRNA name**  circGLIS3 shRNA1(Forward)  circGLIS3 shRNA1(Reverse)  circGLIS3 shRNA2(Forward)  circGLIS3 shRNA2(Reverse) | GGAAAGGCUUAUAACCCACTT GUGGGUUAUAAGCCUUUCCTT UUCUCCGAACGUGUCACGUTT  ACGUGACACGUUCGGAGAATT  **Sequence (5’-3’)**  GATCCCCGGAAAGGCTTATAACCCACTCAAGAGGTGGGTTATAAGCCTTTCCTTTTT  AATTAAAAAGGAAAGGCTTATAACCCACCTCTTGAGTGGGTTATAAGCCTTTCCGGG  GATCCCCGCCTGGGAAAGGCTTATAACTCAAGAGGTTATAAGCCTTTCCCAGGCTTTTT  AATTAAAAAGCCTGGGAAAGGCTTATAACCTCTTGAGTTATAAGCCTTTCCCAGGCGGG |

**
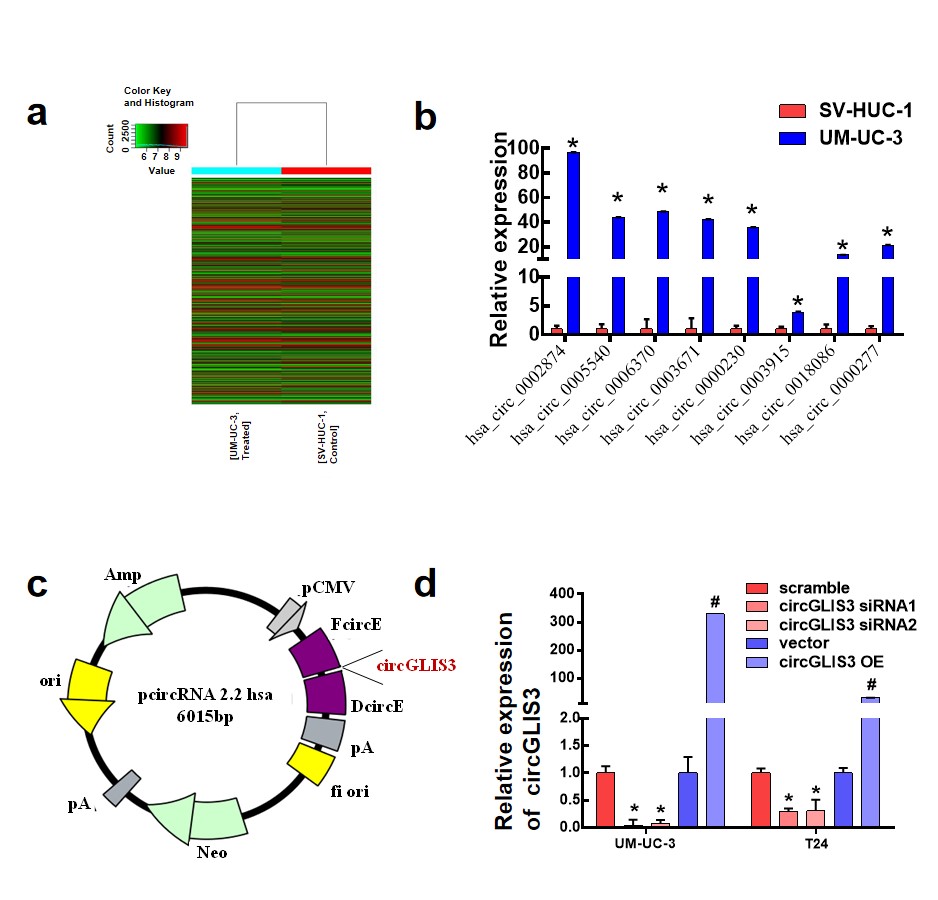
Fig. S1 a** Chip analysis of the circRNA expression profile in bladder cancer cells using SV-HUC-1 as the control group and UMUC3 as the experimental group. **b** has_circ_0002874 gene selection process. We screened the eight circRNAs that were most significantly upregulated in the circRNA differential expression profile of bladder cancer cells, designed primers across the splice site, and verified them using qPCR. We found that circGLIS3 was most significantly increased in the bladder cancer cells **c** circGLIS3-specific overexpression transient vector. **d** Efficiency verification of transient silence and overexpression of circGLIS3. (p< 0.05).
